# Combination of Exercise Testing Criteria to Diagnose Lower Extremity Peripheral Artery Disease

**DOI:** 10.3389/fcvm.2021.759666

**Published:** 2021-11-17

**Authors:** Olivier Stivalet, Anita Paisant, Dihia Belabbas, Alexis Le Faucheur, Philippe Landreau, Estelle Le Pabic, Loukman Omarjee, Guillaume Mahé

**Affiliations:** ^1^Vascular Medicine Unit, CHU Rennes, Rennes, France; ^2^Radiology Department, CHU Rennes, Rennes, France; ^3^University of Rennes 2, M2S-EA 7470, Rennes, France; ^4^Institut National de la Santé Et de la Recherche Médicale (INSERM), CIC1414, Rennes, France; ^5^Vascular Medicine, Centre Hospitalier de Redon, Redon, France; ^6^University of Rennes 1, Rennes, France

**Keywords:** peripheral artery disease (PAD), exercise test, ankle brachial index (ABI), transcutaneous oxygen pressure (TcPO2), claudication

## Abstract

**Objectives:** Nothing is known about the interest of the combination of exercise tests to diagnose Lower-extremity Peripheral Artery Disease (LEPAD). The aim of this study was to assess if combining exercise testing criteria [post-exercise Ankle-Brachial Index (ABI) + exercise-oximetry (exercise-TcPO2)] improves the detection of lower limbs arterial stenoses as compared with post-exercise ABI using American Heart Association (AHA) criteria, or exercise-TcPO2 alone.

**Material and Methods:** In a prospective monocentric study, consecutive patients with exertional-limb pain and normal resting-ABI referred to our vascular center (Rennes, France) were assessed from May 2016 to February 2018. All included patients had a computed tomography angiography (CTA), a resting-ABI, a post-exercise ABI and an exercise-TcPO2. AHA post-exercise criteria, new validated post-exercise criteria (post-exercise ABI decrease ≥18.5%, post-exercise ABI decrease <0.90), and Delta from Rest of Oxygen Pressure (Total-DROP) ≤-15mmHg (criterion for exercise-TcPO2) were used to diagnose arterial stenoses ≥50%. For the different combinations of exercise testing criteria, sensitivity or specificity or accuracies were compared with McNemar's test.

**Results:** Fifty-six patients (mean age 62 ± 11 years old and 84% men) were included. The sensitivity of the combination of exercise testing criteria (post-exercise ABI decrease ≥18.5%, or post-exercise ABI decrease <0.90 or a Total-DROP ≤-15mmHg) was significantly higher (sensitivity = 81% [95% CI, 71–92]) than using only one exercise test (post-exercise AHA criteria (sensitivity = 57% [43–70]) or exercise-TcPO2 alone (sensitivity = 59% [45–72]).

**Conclusions:** Combination of post-exercise ABI with Exercise-TcPO2 criteria shows better sensitivity to diagnose arterial stenoses compared with the AHA post-exercise criteria alone or Exercise-TcPO2 criteria used alone. A trend of a better accuracy of this combined strategy was observed but an external validation should be performed to confirm this diagnostic strategy.

## Introduction

Lower Extremity Peripheral Artery Disease (LEPAD) affects more than 235 million people worldwide (1). The ankle-brachial index at rest (Resting-ABI) is a clinical means recommended by the guidelines of the American Heart Association (AHA) to diagnose the presence and severity of LEPAD regardless of the symptoms reported by the patients (2).

In patients with exertional limb pain relieved by rest and a resting-ABI >0.90, exercise testing with post-exercise ABI measurements is recommended by the AHA (2). The AHA statement proposed two different post-exercise criteria to diagnose LEPAD: either a post-exercise ABI decrease >20% or a post-exercise pressure decrease >30 mmHg. Exercise oximetry (exercise-TcPO2) has also been proposed to diagnose lower limbs arterial stenoses (3–5). Several studies have shown that exercise-TcPO2 using the Delta from Resting Oxygen Pressure (DROP) value is accurate to diagnose arterial stenoses of ≥50% assessed by computed tomography angiography (CTA) or angiography as a gold standard (3–5). Aday et al. has shown that post-exercise ABI < 0.90 shows a better sensitivity than each of the AHA criteria taken separately (6). We have previously found that in patients with a normal resting-ABI, cut off values of post-exercise ABI decrease ≥18.5% or DROP ≤-15 mmHg have similar area under the curves (AUC) to detect LEPAD (i.e., arterial stenoses≥50%) (7). AUCs for post-exercise ABI decrease ≥18.5% and DROP ≤-15 mmHg were 0.67[0.53–0.78] and 0.67[0.53–0.78] respectively (7). We and others have previously demonstrated that discrepancies for the diagnosis of LEPAD exist between exercise criteria (Ankle pressure, post-exercise ABI and exercise-TcPO2) (8–10). Indeed, using a criterion patient can be considered as a LEPAD patient whereas using another criterion the same patient can be considered as a patient without LEPAD.

To date, nothing is known about the interest of the combination of exercise testing criteria to diagnose LEPAD in clinical practice. We hypothesize that the combination of exercise testing criteria could improve the detection of LEPAD in patients with exertional limb symptoms and a resting-ABI >0.90. The aim of this study was to assess if the sensitivity of the combination of exercise testing criteria is higher than the sensitivity of post-exercise ABI using the AHA criteria, or exercise-TcPO2 alone in patients with exertional limb symptoms and a resting-ABI > 0.90.

## Methods

### Ethical Standards

The study was conducted from May 2016 to February 2018 and approved by an institutional review board (IRB) from the University Hospital of Rennes (ref.17.12). All participants gave written informed consent. The study protocol conforms to the ethical guidelines of the 1975 Declaration of Helsinki. The Exercise Peripheral Artery Disease (PAD) study was registered with the American National Institutes of Health database under reference n° NCT03186391.

### Study Design

This is a monocentric study on consecutive patients referred to our vascular unit (University Hospital, Rennes, France) for exertional limb pain suspected of LEPAD. In this population, we selected patients with at least one limb with a resting-ABI >0.90, who had a CTA performed within 3 months of the exercise appointment. In our clinical practice, we systematically perform an exercise-TcPO2 test immediately followed by a second exercise test to measure the post-exercise ankle pressure and post-exercise ABI. Patients who were unable to walk on a treadmill or suffering from heart disease contraindicating an exercise test were not included.

### Patient Demographic Characteristics

Variables collected included age, gender, body mass index, comorbidities, and medications (statins, anti-hypertension treatment, antiplatelet, antidiabetic oral treatment, or insulin).

There were 15 patients (17 limbs with an ABI >0.90) who had undergone peripheral artery procedures before our study such as bypass, stent, or angioplasty on the limb included in the evaluation.

### ABI Measurement

After a careful clinical evaluation, a measurement of resting-ABI was performed according to AHA recommendations (2) using a hand-held Doppler probe (8 MHz; Basic Atys Medical™, Soucieu en Jarrest, France) by a trained vascular medicine physician. Briefly, the patient was at rest for 10 min in the supine position, relaxed, head and heels supported, in a room with a comfortable temperature (21°C) (11). The following counterclockwise sequence was used for the systolic arterial pressure measurement: “right brachial artery, right posterior tibial artery, right dorsalis pedis artery, left posterior tibial artery, left dorsalis pedis artery, left brachial artery, and right brachial artery” as mentioned by AHA Guidelines (2). The resting-ABI was calculated by dividing the highest pressure of the limb (dorsalis pedis or posterior tibial pressures) by the highest arm pressure as recommended (12). For the brachial artery, contrary to the AHA guidelines, we used an automatic blood pressure monitor (Carescape™ Dinamap V100; GE Healthcare) in order to have the same procedure to measure the pressure at rest and after exercise (7).

### Treadmill Test

A treadmill walking test (3.2 km/h, 10% slope) was used up to a maximal distance of 1053m (20 min). This test was used for both the exercise-TcPO2 measurement, which was performed first, and for the post-exercise pressure measurements. A minimal recovery period of 10 min was required between the two exercise tests. The patients were asked to inform the physician when and where (buttock, thigh, calf, or other) the pain appeared during the test. Exercise was stopped for both tests according to the limitation of the patient.

### Exercise-TcPO2 Measurement

Briefly, measurement of TcPO2 was performed using calibrated TcPO2 electrodes (TCOM/TcPO2; PF 6000TcPO2/CO2 Unit; Perimed; Jarfalla, Sweden). A reference electrode (chest electrode) was placed between the scapulae and the spine to measure systemic changes in TcPO2 during exercise (3, 13, 14). One electrode was positioned on each buttock, 4 to 5 cm behind the bony prominence of the trochanter, and one electrode on each calf (3, 13, 14). Exercise was performed on a treadmill at a 10% slope and a speed of up to 3.2 km/h (13). Exercise was discontinued at the request of the patient or, by protocol, up to a maximum exercise duration of 20 min. The measurement from the TcPO2 electrodes was used to calculate the Delta from Resting Oxygen Pressure (DROP) index, which was expressed in mmHg (14). We define Total DROP as the lowest DROP value between proximal and distal electrodes on each limb. DROP was recorded in real-time by the in-house Oxymonitor (version 2019.01.05) free Software (https://imagemed.univ-rennes1.fr/en/oxymonitor/download.php) as previously described (15). As defined in a previous study, we considered a Total DROP ≤-15 mmHg accurate to diagnose arterial stenoses of ≥50% assessed by computed tomography angiography (CTA) as a gold standard (7).

### Post-exercise Pressure and Post-exercise ABI Measurements

Two persons performed the measurements: one at the brachial level with the automatic blood pressure device (Carescape™ Dinamap V100; GE Healthcare) and one at the limb level with the handheld Doppler (7, 16).

Post-exercise pressures were assessed on the same arteries as it was for the resting-ABI measurement (16). When the resting-ABI was measured, a black pen was used to mark the skin area where the highest limb pressure had been recorded with a hand-held Doppler. Following exercise, we were sure that we were in the correct area to perform the post-exercise pressure measurement and if there was no arterial flow it meant that the pressure was 0 mmHg (17). The highest ankle pressure of each limb at rest was assessed, beginning with the more symptomatic limb. Post-exercise pressures were assessed within 1 min after the termination of walking (18).

### Arterial Stenoses Quantification Using CTA

Computed tomography angiography was performed in all subjects within 3 months before or after the post-exercise ABI and exercise-TcPO2 measurements to confirm the arterial stenotic lesions. The methodology is similar to the one previously published by our team (7). CTA was performed with a 64-slice CT scanner (Discovery CT 750 High Definition; GE Healthcare, Milwaukee, WI, USA), 100-kV tube voltage, and an automatic modulation of mAs (80–500 mAs). The scanning range was planned with a scout view and included the entire vascular tree from the abdominal aorta to ankles. A total of 120 ml or 1.5 ml/kg of iobitridol 350 mgI/ml (Xenetix®, Guerbet, Roissy, France) was administered with an automated injector at a flow rate of 4 ml/sec. There was systematically a 3D MIP reconstruction (Maximum Intensity Projection) and a 2D multiplanar reconstruction (MPR). CTA data were transferred to a computer workstation (Advantage Workstation, AW 4.6; GE Medical Systems) for analysis. The reformatted 1.25-mm axial images, multiplanar reformats and Vessel Analysis© software (GE Healthcare, Milwaukee, WI, USA) were used to determine the grade of stenosis. The referring doctor of the patient ordered CTA at his or her discretion. CTA, used as gold standard, was performed to detect luminal arterial stenoses in each patient in our facility. Significant stenoses (≥50% of the diameter) at each artery level (aorta, common iliac artery, external iliac artery, internal iliac artery, common femoral artery, superficial femoral artery, popliteal artery on both sides) were reported by two blinded radiologists (AP and DB) who were unaware of the results of the exercise tests appointment. Infrapopliteal artery stenoses were not assessed. In case of variability higher than 10% for ≥50% stenoses between the two radiologists, a new interpretation was performed with both. The percent stenoses were calculated as follows by each physician: 100 x [1 – (diameter of the lumen at the site of the stenosis/diameter of the normal lumen)]. Finally, the degree of stenoses at each artery level used for the statistical analyses was calculated as the mean of the quantification performed by both physicians or, in the case of a third interpretation, a third measurement was used.

### Data Analysis

Post-exercise pressure decrease, post-exercise ABI were calculated and results of exercise-TcPO2 were analyzed without knowing the results of CTA. Right Total DROP allows the detection of flow-reducing lesions in the following arteries: the aorta, the right common iliac artery, the right external iliac artery, the right common femoral artery, the right superficial femoral artery, and the right popliteal artery. Left Total DROP allows the detection of flow-reducing lesions in the following arteries: the aorta, the left common iliac artery, the left external iliac artery, the left common femoral artery, the left superficial femoral artery, and the left popliteal artery (14).

### Statistical Analyses

Results are expressed as mean ± standard deviation. Continuous variables were expressed as mean and 95% confidence interval (CI), and categorical variables as numbers (percentages). Then, sensitivity, specificity, positive predictive value, and negative predictive value were calculated with their respective 95% CI. For the different combinations of exercise testing criteria, sensitivity, or specificity or accuracies were compared with McNemar's test. Statistical analyses were performed with SAS software, v.9.4® (SAS Institute, Cary, NC, USA). For all statistical tests, a two-tailed probability level of *p* ≤ 0.05 was used to indicate statistical significance.

## Results

**Figure 1:**
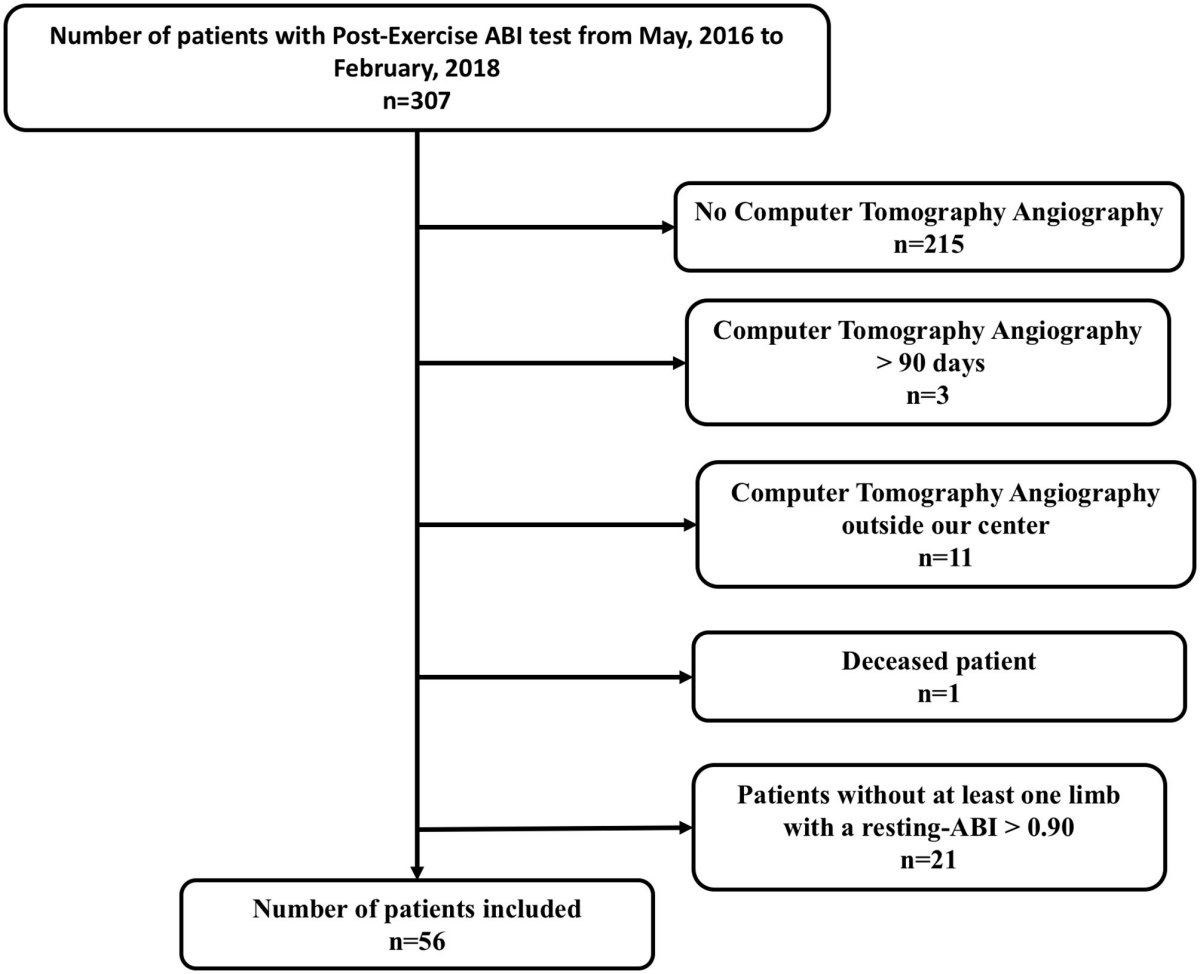
Flow chart. ABI, Ankle-Brachial Index.

Among 307 patients, 56 patients (83.9% men) met all criteria (at least one limb with ABI > 0.90 and exertional limb pain and CTA perform within 3 months) to be included in this prospective study (Figure 1). Characteristics of patients are presented in Table 1. The average age and body mass index were 62 ± 11 years old, 26.5 ± 5.1 kg/m^2^, respectively. Among the limbs with a resting-ABI > 0.90 (*n* = 86), 53 limbs had arterial stenoses. Among these 53 limbs, 85 stenoses were identified as follows: 3 on the aorta, 22 on the common iliac artery, 4 on the external iliac artery, 23 on the internal iliac artery, 3 on the common femoral artery, 18 on the superficial femoral, and 12 on the popliteal artery.

**Table 1:** Population characteristics.

**Clinical characteristics**	***n*** **= 56**
Age, y, mean ± standard deviation	62 ± 11
Male sex, no., (%)	47 (83.9%)
Body mass index, kg/m^2^, mean ± standard deviation	26.5 ± 5.1
**Comorbidities, (history of), no. (%)**	
Current smoker, no. (%)	21 (37.5%)
History of smoking, no. (%)	45 (80.4%)
Hypercholesterolemia, no. (%)	34 (60.7%)
Diabetes, no. (%)	10 (17.9%)
Peripheral artery vascular surgery^*^, no. (%)	19 (33.9%)
Hypertension, no. (%)	37 (66.1%)
History of coronaropathy, no. (%)	17 (30.4%)
History of stroke, no. (%)	9 (16.1%)
**Current medications, no. (%)**	
Statins, no. (%)	32 (57.1%)
Anti-Hypertension treatments, no. (%)	38 (67.8%)
Antiplatelets, no. (%)	42 (75%)
Anti-diabetic oral treatments, no. (%)	8 (14.3%)
**Resting Ankle-Brachial index**	
Resting ankle-brachial index (right), mean [CI 95%]	1.03 [0.78–1.28]
Resting ankle-brachial index (left), mean [CI 95%]	0.95 [0.74–1.16]
**Indication of the treadmill test**, **no. (%)**	
Exertional limb pain, no. (%)	52 (92.9%)
Muscle fatigability (limbs), no. (%)	4 (7.1%)

* *Peripheral artery Vascular surgery from aorta and/or more distal iliac or leg arteries*.

**Figure 2:**
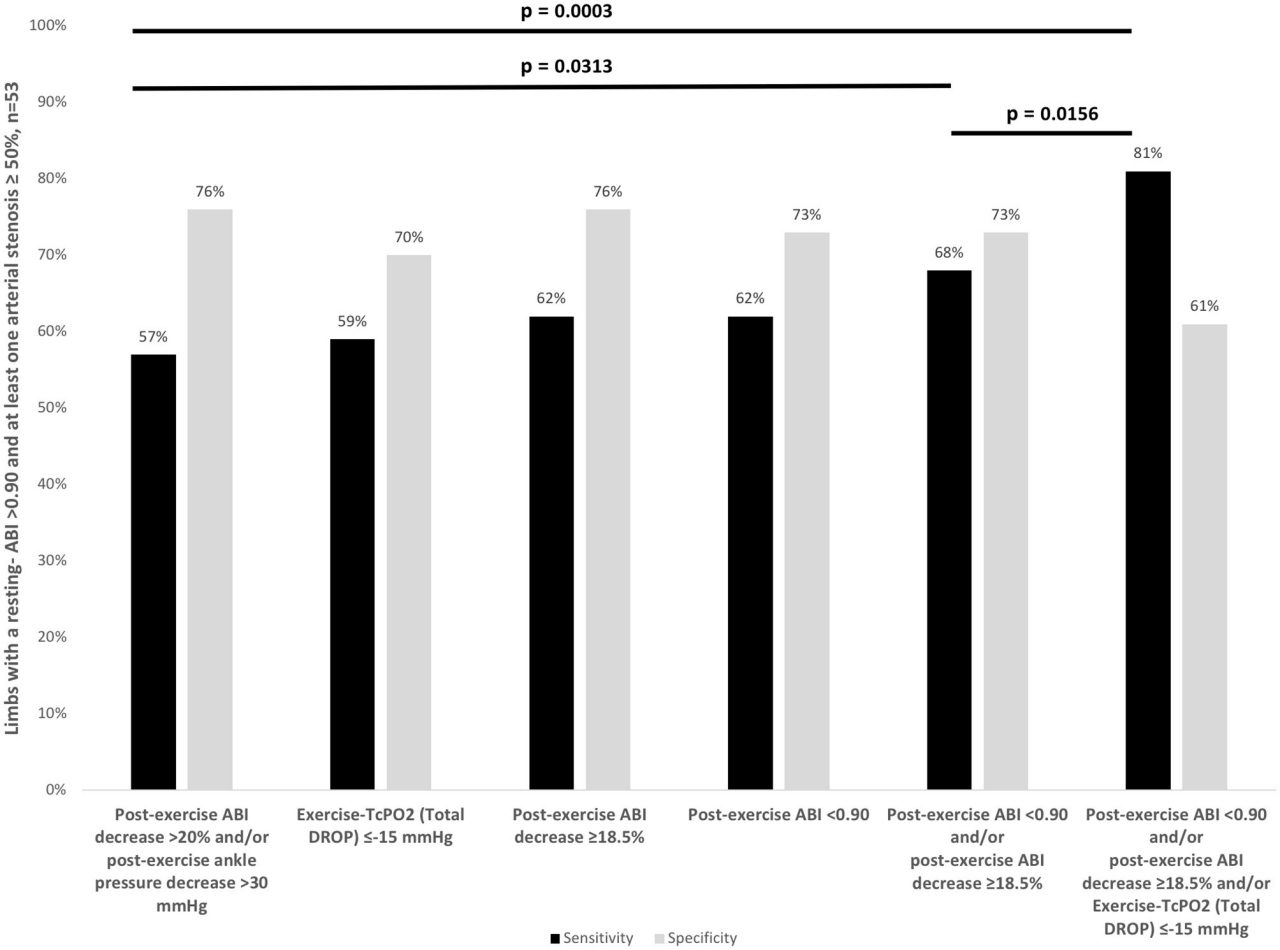
Sensitivity and specificity of exercise tests criteria to diagnose Lower Extremity Peripheral Artery Disease (LEPAD) in patients with an ABI >0.90 and at least one arterial stenosis ≥50%. CI, confidence interval; ABI, Ankle-Brachial Index; DROP, Delta from Resting Oxygen Pressure.

Among limbs with a normal resting-ABI (*n* = 86), Table 2 shows the results of the different exercise tests to detect limbs of patients with or without arterial stenoses ≥50% detected with CTA. Figure 2 shows for limbs of patients with a resting-ABI >0.90 and an arterial stenosis ≥50% (*n* = 53) along with the proportion of patients' limbs with arterial stenoses detected by the different criteria or combinations of criteria.

**Table 2:** Diagnosis performances of the different exercise criteria to diagnose lower extremity peripheral artery disease.

**Population (n = 56)** **Limbs with a resting-ABI >0.90 (n = 86)**	**Sensitivity (95%CI)**	**Specificity (95%CI)**	**Positive predictive value (95%CI)**	**Negative predictive value (95%CI)**	**Accuracy (95%CI)**
Post-exercise ABI decrease >20% *or* Post-exercise ankle pressure decrease >30 mmHg	57% [43–70]	76% [61–90]	79% [66–92]	52% [38–66]	64% [54–74]
Exercise-TcPO2 (Total DROP) ≤-15 mmHg	59% [45–72]	70% [54–85]	76% [63–89]	51% [37–66]	63% [53–73]
Post-exercise ABI decrease ≥18.5%	62% [49–75]	76% [61–90]	81% [68–93]	56% [41–70]	67% [57–77]
Post-exercise ABI <0.90	62% [49–75]	73% [58–88]	79% [66–91]	55% [40–69]	66% [56–76]
Post-exercise ABI <0.90 *or* Post-exercise ABI decrease ≥18.5%	68% [55–81]	73% [58–88]	80% [68–92]	59% [44–74]	70% [60–80]
Post-exercise ABI decrease >20% *or* Post-exercise ankle pressure decrease >30 mmHg *or* Exercise-TcPO2 (Total DROP) ≤-15 mmHg	74% [62–86]	64% [47–80]	77% [65–88]	60% [44–76]	70% [60–80]
Post-exercise ABI decrease ≥18.5% *or* Exercise-TcPO2 (Total DROP) ≤-15 mmHg	77% [66–89]	64% [47–80]	77% [66–89]	64% [47–80]	72% [63–82]
Post-exercise ABI <0.90 *or* Exercise-TcPO2 (Total DROP) ≤-15 mmHg	77% [66–89]	61% [44–77]	76% [65–87]	63% [46–79]	71% [61–81]
Post-exercise ABI <0.90 *or* Post-exercise ABI decrease ≥18.5% *or* Exercise-TcPO2 (Total DROP) ≤-15 mmHg	81% [71–92]	61% [44–77]	77% [66–88]	67% [50–84]	73% [64–83]

*ABI, Ankle-Brachial Index; TcPO2, Transcutaneous Oxygen Pressure measurements; DROP, Delta from Rest Oxygen Pressure; CI, Confidence Interval*.

We show that the sensitivity of the combination of exercise criteria (post-exercise ABI decrease ≥18.5%, or post-exercise ABI < 0.90 or a Total DROP ≤-15 mmHg) is greater than the sensitivity of the AHA criteria (post-exercise ABI decrease >20% or post-exercise pressure >30 mmHg) or the sensitivity of exercise-TcPO2 alone.

The sensitivity, specificity, positive and negative predictive values (95% CI), and accuracies are presented in Tables 2, 3. Globally, the increase of the number of exercise criteria increases the accuracy of the tests. The highest accuracy is found for the combination of three exercise criteria (post-exercise ABI decrease ≥18.5%, or post-exercise ABI < 0.90 or a Total DROP ≤-15mmHg). This accuracy is statistically better than the accuracy of post exercise pressure >30 mmHg (*p* = 0.0035) and Exercise TcPO2 criterion (*p* = 0.0201). A trend to an improvement of the accuracy was also observed as compared with the combination of post-exercise ABI decrease >20% or post-exercise ankle pressure decrease >30 mmHg (*p* = 0.0593).

**Table 3:** Comparisons of the accuracies of the different tests.

**Accuracy**								
**Post-exercise ABI <0.90** ***or*** **post-exercise ABI decrease ≥18.5% or exercise-TcPO2 (Total DROP) ≤-15 mmHg**	**Post-exercise ABI <0.90**	**Post-exercise ABI decrease >20%**	**Post-exercise ankle pressure decrease >30 mmHg**	**Post-exercise ABI decrease ≥18.5%**	**Exercise-TcPO2 (Total DROP) ≤-15 mmHg**	**Post-exercise ABI decrease >20%** ***or*** **post-exercise ankle pressure decrease >30 mmHg**	**Post-exercise ABI <0.90** ***or*** **post-exercise ABI decrease ≥18.5%**	***p*** **value**
73% [64–83]	66% [56–76]							0.1088
73% [64–83]		64% [54–74]						0.0593
73% [64–83]			52% [42–63]					0.0035
73% [64–83]				67% [57–77]				0.1967
73% [64–83]					63% [53–73]			0.0201
73% [64–83]						64% [54–74]		0.0593
73% [64–83]							70% [60–80]	0.3657

*ABI, Ankle-Brachial Index; DROP, Delta from Resting Oxygen Pressure*.

There were discrepancies between AHA criteria (post-exercise ABI decrease >20% or post-exercise ankle pressure decrease > 30 mmHg) and the combination of post-exercise ABI with Exercise-TcPO2. The characteristics of patients are described in Table 4. Ten patients (12 limbs) who had arterial stenosis ≥50% had discrepancies between AHA criteria (i.e., post-exercise ABI decrease >20% or post-exercise ankle pressure decrease >30 mmHg) and combination of post-exercise ABI with Exercise-TcPO2 [post-exercise ABI < 0.90 or post-exercise ABI decrease ≥18.5% or exercise-TcPO2 (Total DROP) ≤-15 mmHg]. Five out of twelve limbs (42%) had stenosis localized at the internal iliac artery level. Only one patient (1 limb) stopped the test due to exertional limb pain localized on the limb with an arterial stenosis ≥50%. Seven patients stopped for pain on both limbs (3 patients/5 limbs) or pain on the other limb (4 patients/4 limbs). The last two patients (2 limbs) were not limited on the treadmill (1053m). There was no case of a limb considered as diseased with the AHA criteria (i.e., post-exercise ABI decrease >20% or post-exercise ankle pressure decrease >30 mmHg) and not diseased with the combination of post-exercise ABI with Exercise-TcPO2 criteria.

**Table 4:** Characteristics of patients of which there were discrepancies between the American Heart Association (AHA) post-exercise criteria (negative for the diagnosis) and the combination of post-exercise ankle-brachial index (ABI) with Exercise oximetry (TcPO2) (positive for the diagnosis).

**Limbs with a resting ABI > 0.90 and an arterial stenosis ≥50%**	**Gender**	**Age (years)**	**Location of arterial stenosis ≥50%**	**Resting-ABI of the limb**	**Diabetes**	**Vascular surgery**	**MWD (meter) first test**	**Cause of stopping for first test**	**MWD (meter) second test**	**Cause of stopping for second test**
1	Woman	74	Aorta	0.96	No	No	1,053	None	1,053	None
2	Woman	60	Aorta	0.97	No	No	1,053	None	1,053	None
3	Man	49	CIA	0.91	No	Yes	208	Exertional contralateral limb pain	211	Exertional contralateral limb pain
4	Man	67	CIA	1.05	No	No	61	Exertional both limbs pain and dyspnea	59	Exertional both limbs pain and dyspnea
5	Man	69	IIA, CIA, CFA, SFA, PA	1.47	Yes	No	285	Exertional both limbs pain	330	Exertional both limbs pain
6	Man	69	IIA, CIA, CFA, SFA, PA	1.53	Yes	No	285	Exertional both limbs pain	330	Exertional both limbs pain
7	Man	44	IIA	1.15	No	No	108	Exertional contralateral limb pain	110	Exertional contralateral limb pain
8	Man	58	IIA	0.94	No	No	205	Exertional both limbs pain	198	Exertional both limbs pain
9	Man	58	IIA, CIA, CFA, SFA, PA	0.94	No	No	205	Exertional both limbs pain	198	Exertional both limbs pain
10	Man	65	SFA	0.93	No	No	345	Exertional contralateral limb pain	284	Exertional contralateral limb pain
11	Man	77	SFA	1.19	No	No	133	Exertional limb pain	218	Exertional limb pain
12	Man	49	SFA, PA	1.07	No	No	244	Painful sore on the contralateral limb	389	Exertional both limbs pain

*CIA, common iliac artery; IIA, internal iliac artery; CFA, common femoral artery; SFA, superficial femoral artery; PA, popliteal artery; MWD, Maximal walking distance; Vascular surgery, graft, angioplastia or stenting. Joined lines in blue correspond to a same patient*.

Nine patients (10 limbs) were identified neither by the AHA criteria nor by the combination of post-exercise ABI with Exercise-TcPO2 (post-exercise ABI < 0.90 or post-exercise ABI decrease ≥18.5% or exercise-TcPO2 (Total DROP) ≤-15 mmHg). The characteristics of patients are described in Table 5. Four patients (5 limbs) were not limited on the treadmill test (1053m). Three patients (3 limbs) stopped due to contralateral pain and two stopped due to fatigue (1 patient/1 limb) and vertigo (1 patient/1 limb).

**Table 5:** Characteristics of patients of which neither AHA post-exercise criteria nor the combination of post-exercise ABI with Exercise-TcPO2 detect arterial stenosis ≥50%.

**Limbs with a resting ABI > 0.90 and an arterial stenosis ≥50%**	**Gender**	**Age (years)**	**Location of arterial stenosis ≥50%**	**Resting-ABI of the limb**	**Diabetes**	**Vascular surgery**	**MWD (meter) first test**	**Cause of stopping for first test**	**MWD (meter) second test**	**Cause of stopping for second test**
1	Man	63	CIA	1.06	No	No	478	Vertigo	1,053	None
2	Man	70	CIA	1.05	No	No	1,053	None	1,053	None
3	Man	70	CIA	0.99	No	No	1,053	None	1,053	None
4	Man	84	CIA, SFA	0.98	Yes	No	285	Dyspnea	254	Dyspnea
5	Man	58	CIA, SFA	1.13	No	No	235	Exertional contralateral limb pain	333	Exertional both limbs pain
6	Man	72	IIA	1.02	No	No	1,053	Exertional contralateral limb pain	1,053	Exertional contralateral limb pain
7	Woman	76	IIA	1.05	No	No	98	Exertional contralateral limb pain	98	Exertional contralateral limb pain
8	Man	51	SFA	1.96	No	No	1,053	None	1,053	None
9	Man	82	PA	1.66	No	No	281	Exertional contralateral limb pain	358	Exertional contralateral limb pain
10	Man	77	PA	1.12	Yes	No	93	General fatigue	68	General fatigue

*CIA, common iliac artery; IIA, internal iliac artery; CFA, common femoral artery; SFA, superficial femoral artery; PA, popliteal artery; MWD, Maximal walking distance; Vascular surgery, graft, angioplastia or stenting. Joint lines in blue correspond to a same patient*.

## Discussion

To our knowledge, this is the first study designed to evaluate the diagnostic value of a combination of exercise testing criteria including post-exercise ABI and exercise-TcPO2 criteria to detect arterial stenoses ≥50% in patients with normal resting-ABI. This study shows that the sensitivity of the combination of exercise criteria (post-exercise ABI decrease ≥18.5%, or post-exercise ABI < 0.90 or a Total DROP ≤ −15 mmHg) is statistically better than the sensitivity of the AHA criteria (post-exercise ABI decrease >20%, or post-exercise pressure >30 mmHg) or the sensitivity of exercise-TcPO2 alone.

For patients with a normal resting-ABI suspected of LEPAD, the AHA recommends to perform a post-exercise ABI using a post-exercise ABI decrease >20%, and/or a post-exercise pressure > 30 mmHg as criteria to diagnose LEPAD (Class IIa; Level of Evidence A) (2). A previous study had shown a dissonance between these two criteria (19). Previous studies about the accuracy of exercise tests to diagnose LEPAD were conducted in the 80s and suffer from many bias (7, 19–21). Therefore, in a previous paper, we defined new criteria of post-exercise ABI (post-exercise ABI decrease ≥18.5%) and exercise-TcPO2 criterion (Total DROP ≤-15 mmHg) with a current treadmill test (3.2 km/h and 10% grade) performed in clinical routine to detect significant arterial stenosis (7). In the meantime, Aday et al. has confirmed that post-exercise ABI < 0.90 was also a good candidate to diagnose LEPAD (6, 7). In case of incompressible lower limb arteries, the resting-ABI and post-exercise ABI can be falsely reassuring. In this context, the exercise-TcPO2 can be of interest (14, 22). In this study, we show for the first time that a combination of exercise tests improves diagnosis of arterial stenoses ≥50% in patients suspected of having LEPAD (i.e., with exertional limb symptoms) with normal resting-ABI.

Some authors might think that there is no interest to use several exercise tests for cost and time issues, but our study demonstrates a synergy between both exercise tests. Furthermore, performing these two exercise tests might finally reduce the number of CTA that are performed to find the etiology of the exertional limb symptoms.

In our study, the sensitivity is higher for the combination of the new exercise criteria (post-exercise ABI decrease ≥18.5%, or post-exercise ABI < 0.90) with exercise-TcPO2 [81%(71–92)] or without exercise-TcPO2 [68%(55–81)] than the sensitivity of AHA criteria (post-exercise ABI decrease >20%, or post-exercise pressure >30mmHg) [57%(43–70)] with a slightly lower specificity [73%(58–88) vs. 76%(61–90) for AHA criteria] without exercise-TcPO2 and a lower specificity with exercise-TcPO2 [61% (44–77)]. The AHA criteria (post-exercise ABI decrease >20%, or post-exercise pressure >30 mmHg) and the exercise-TcPO2 (Total DROP ≤-15 mmHg) seems to have similar sensitivity [57%(43–70) and 59%(45–72)], but specificity of exercise-TcPO2 is slightly lower [70%(54–89%) vs. 76%(61–90) for AHA criteria]. To comfort our results and study the statistical significance, an external validation remains to be performed. Of interest, the sensitivity of post-exercise ABI decrease >20% [57%(43–70)] was significantly better than the sensitivity of post-exercise pressure >30mmHg [28%(16–40), 95%CI] confirming previous suggestions that the use of a post-exercise ankle pressure decrease > 30 mm Hg to diagnose PAD should not be proposed anymore (23).

Unfortunately, the combination of post-exercise ABI with exercise-TcPO2 did not detect all limbs with an arterial stenosis ≥50% (i.e., 18.8% limbs were missed). Of interest, in none of these cases, patients stopped the treadmill due to exertional limb symptom on the limb with an arterial stenosis ≥50%. This suggests that to be informative, exercise tests and their diagnosis criteria have to be symptom-limited to be accurate as suggested in previous papers (24–26).

Based on the literature and our results, we suggest in order to diagnose LEPAD in patients with exertional limb symptoms that first, the physician should perform resting-ABI. Second, in case of resting-ABI > 0.90, physicians should perform post-exercise ABI measurements or exercise-TcPO2 on a treadmill test of 3.2km/h and 10% grade with the following cut-offs: post-exercise ABI decrease of ≥18.5%; post-exercise ABI <0.90; and Total DROP index of ≤-15mmHg (7). Third, if the test is normal, a second exercise testing should be performed. This could either be post-exercise ABI if exercise-TCPO2 was done first. or exercise-TcPO2 if post-exercise ABI was performed first.

### Limits

Our study has several limitations. First, we used an automatic blood pressure monitor to assess brachial blood pressure at rest and after exercise. We know that the AHA guidelines recommend performing all pressure measurements with a handheld Doppler at rest (2). However, our objective was that this new diagnosis strategy can be used in clinical practice where in most cases, only one person is devoted to perform the measurement. In that case and based on previous work from Gardner and Montgomery, we have decided to use an automatic blood pressure measurement to perform as quickly as possible the different pressure measurements because pressure can return to normal value very quickly in some patients (27). In order to avoid any bias, we applied the same method at rest. Second, it was not possible to assess the reproducibility of the different tests. However, our reproducibility to perform resting-ABI and the reproducibility of exercise-TcPO2 have been previously reported as good (20, 28). Third, characterization of stenoses has been made with CTA rather than Doppler ultrasound (DUS) in order to allow a double reading of the CTA rather than one reading/operator with the DUS. In a meta-analysis, the reported sensitivity and specificity of CTA to detect aorto-iliac stenoses >50% were 96 and 98%, respectively, with similar sensitivity (97%) and specificity (94%) for the femoropopliteal region (29). Digital subtraction angiography was not retained as a gold standard due to its invasive nature. Fourth, we decided to consider the presence of LEPAD according to the presence of at least one arterial stenosis ≥50% according to the most common practice in the literature (30–37). Fifth, we assess the presence of stenoses on the aorto-iliac and femoro-popliteal tract without studying infrapopliteal arteries. Indeed, we decided not to explore the distal arterial axis due to: (i) the absence of an evaluation of the 3 arterials axis by the resting-ABI (posterior and anterior tibial artery only), (ii) an arterial stenosis ≥50% on one calf arterial axis could not formally be correlated to an abnormal resting-ABI and formally linked to an intermittent claudication, and iii) degree of infra-popliteal artery stenoses is not assessed, only their permeability. Finally, this last limitation is similar for all exercise tests.

## Conclusion

Our study shows that the sensitivity of a combination of post-exercise criteria (post-exercise ABI decrease ≥18.5%, or post-exercise ABI < 0.90) with exercise-TcPO2 (Total DROP ≤-15 mmHg) is significantly improved as compared with the use of AHA criteria (post-exercise ABI decrease >20%, or post-exercise pressure >30 mmHg) alone. A trend of a better accuracy of this combined strategy was observed but an external validation should be performed to confirm this diagnostic strategy.

## Data Availability Statement

The raw data supporting the conclusions of this article will be made available by the authors, without undue reservation.

## Ethics Statement

The studies involving human participants were reviewed and approved by Institutional Review Board (IRB) from the University Hospital of Rennes (ref.17.12). The patients/participants provided their written informed consent to participate in this study.

## Author Contributions

OS and GM: protocol conception and design, data interpretation, and drafting of the paper. OS, PL, LO, and GM: data acquisition. OS, AP, DB, ELP, ALF, and GM: data analysis and writing of the paper. Each author revised the report and approved the submitted version of the manuscript. Each author has agreed both to be personally accountable for his/her own contribution and to ensure that questions related to the accuracy or integrity of any part of the work, even those in which the author was not personally involved, are appropriately investigated, resolved, and the resolved outcome documented in the literature. All authors contributed to the article and approved the submitted version.

## Funding

The Article Processing Charges (APC) were funded by CHU Rennes.

## Conflict of Interest

The authors declare that the research was conducted in the absence of any commercial or financial relationships that could be construed as a potential conflict of interest.

## Publisher's Note

All claims expressed in this article are solely those of the authors and do not necessarily represent those of their affiliated organizations, or those of the publisher, the editors and the reviewers. Any product that may be evaluated in this article, or claim that may be made by its manufacturer, is not guaranteed or endorsed by the publisher.
